# A Study on the Reliability and Validity of the Korean Health Literacy Instrument for Late School-Aged Children

**DOI:** 10.3390/ijerph181910304

**Published:** 2021-09-30

**Authors:** Sook-Kyoung Park, Eun-Gyeong Kim

**Affiliations:** 1 College of Nursing, Jeonbuk National University, Jeonju 54896, Korea; yoursky@jbnu.ac.kr; 2 Department of Nursing, Kunsan National University, Gunsan 54150, Korea

**Keywords:** health literacy, child, health promotion

## Abstract

This study aimed to develop and validate the Korean Health Literacy Instrument, which measures Korean late school-aged children’s understanding capacity. The construct’s concepts were drawn from the literature review and interviews with school nurses and teachers. A survey was then conducted in 552 fifth and sixth graders in nine elementary schools, from 1 to 9 May 2014. The KR-20 coefficient for reliability, difficulty index, discrimination index, item-total correlation, and known group technique for validity were performed. An exploratory factor analysis was performed to test the construct validity of the instrument and its unidimensionality. The results reveal that a two-factor structure was appropriate for the Korean school-age health literacy tool (root mean square error of approximation = 0.06, Comparative Fit Index = 0.96, and Tucker–Lewis Index = 0.95). From the remaining 16 items, the internal consistency reliability coefficient of this instrument was 0.85, and the criterion-related validity was 0.62 (*p* < 0.001). The Korean health literacy instrument for late school-aged children was suitable for screening individuals who have limited health literacy. Based on the findings of this study, future studies must continue to conduct empirical investigations on the Korean health literacy instrument for late school-aged children

## 1. Introduction

Many people are now aware of the importance of the early detection and prevention of illnesses and are interested in increasing their health knowledge to gain a healthier daily life. However, certain people are not able to utilize available health information to prevent illness because of the lack of understanding of health information. Consequently, their activities related to effective health promotion behavior and disease prevention are limited. As such, there is a perception that a lack of health knowledge is not a problem; however, a bigger problem arises if health-related information is not understood. Health literacy is a related concept in this situation. It was a concept introduced in the United States, where the health illiteracy rate was high at the time. It was a term used to describe the ability to understand health-related words, although its definition has changed over time. Eventually, it gained attention as a major factor influencing the medical-related decision-making of those with linguistic disadvantages, such as the less-educated population and immigrants. Furthermore, health literacy was identified as a major predictor of health factors, such as health equity and competency [1]. Health literacy refers to the ability to acquire, process, and understand health information and services for healthcare-related decision-making, communication, and performance of appropriate health behaviors [2]. It has been reported as a major indicator of health levels of individuals, families, communities, and countries; subsequently, improving health literacy is one of the main goals of Health Plan 2020 in the United States [3,4].

People with low levels of health literacy often fail to perform activities for disease management or health promotion, because of the lack of understanding of disease, resulting in repeated hospitalizations, which, in turn, causes overspending regarding medical expenses. Studies that develop tools for measuring the health literacy of vulnerable groups, such as low-income, less-educated, elderly, and migrant populations, who are considered as having low health literacy [5,6], have been conducted globally [7,8,9,10]. Studies on the level of and factors influencing health literacy [10,11] have also been carried out. However, the latter studies have focused mainly on adults with low health literacy in the hospital system and local community. Accordingly, there is a lack of study on child health literacy [12,13]. The reason for this lack of study is that the health problems of children are relatively lesser compared with the adults or the elderly; therefore, the utilization rate of medical institutions is low. Moreover, their parents or guardians accompany their children when the latter need medical attention. Furthermore, in Korea, there is a lack of educational content to promote healthy habits in children. Most health-related content for children tends to center on simple activities, such as washing hands, brushing teeth, and not eating junk food. Health literacy refers to the ability to accurately understand and interpret health information needed to lead a healthy life. In particular, healthy literacy is developed through childhood education, and sound health literacy developed during this period will help an individual to lead a healthy life as an adult. Additionally, as the concept of adult health literacy has changed in recent years and the number of children requiring chronic health management is increasing, due to the rise in the survival rate of children with chronic diseases related to medical development, children need to understand and must be able to manage their health themselves. Health literacy is also needed to help children grow into healthy adults and perform well in their own health promotion activities. Additionally, in Korea, the number of multicultural families whose first language is not Korean is gradually increasing; thus, children of these families are emerging as a vulnerable group in terms of health literacy. Therefore, to understand and improve the level of children’s health literacy, it is necessary to develop a measurement tool to define and measure the concept of child health literacy. Moreover, the late school age is the most active period for mental and physical development throughout the lifespan; in this stage, emotional independence is gradually achieved, with systematic and continuous development and modifications in the understanding of health and disease. Therefore, this is a very favorable period [14]. The late elementary school years are a period of particular interest due to rapid physical and physiological growth and changes [15], and this is a period of cognitive achievement through concrete concepts and logical thinking. Therefore, [16], it is an appropriate phase to acquire healthy literacy.

Previous studies on children’s health literacy include those conducted in other countries that have constructed a concept of children’s health literacy and a theoretical framework [12,13,17]. Two instruments for measuring health literacy for primary school (9–13 years) students have been developed [12,18], but there are linguistic limitations to applying these to elementary school students in Korea. In addition, other studies have investigated the relationship between health literacy and health promotion behavior, health attitudes, and self-efficacy in children and adolescents [19], and between health literacy and drinking, drug abuse, and violence behaviors [20]. Several tools have been developed internationally for late school-age children (age: 12–13 years), but most of the research has been conducted with adolescents (age: 15–24 years) and late school-age children (age: 12 years). However, the concept of health literacy in children, the understanding and acquisition of health information, and the ability to use and utilize health information could all be different between adults and children. Although the tools developed abroad can be translated and then used among Korean children, their application is limited, owing to the differences in language expression and culture.

Moreover, the development of a Korean health literacy measurement tool for late school-aged children precedes the program of improving health literacy; it is imperative to develop the program and assess the level of health literacy in late childhood.

## 2. Methods

### 2.1. Study Design

This was a methodological study aimed at examining the reliability and validity of the Korean health literacy instrument for late school-aged children.

### 2.2. Participants and Setting

The subjects were late school-aged children in fifth and sixth grade in nine elementary schools (S City, 1; G province, 2; small cities in J province, 3; and small towns in J province, 3). The students who could read and write without difficulty in school life were selected, with the help of the homeroom teacher and school nurse. The reason for selecting fifth and sixth graders as study subjects is that they are in the stage of cognitive development which includes logical and abstract thinking abilities. At this stage, the evaluation of themselves and the environment becomes clear, meaning that they can express themselves accurately. In addition, it is the time during which they form healthy behaviors based on physical and health knowledge [12]. Therefore, they are the main object of educational interventions related to health. There are various opinions on the sample size of the methodological study. In this study, based on the 2-parameter logistic model (2PLM) of the item response theory, 570 subjects were selected according to the criterion that a minimum of 500 subjects were required based on 30 items [21], and that the number of subjects was more than 500 in the development of the tool [22].

### 2.3. Measurement

#### 2.3.1. Selection of Constituent Factors

To identify the components of health literacy, the preceding studies and existing tools on health literacy of adults, adolescents, and children were reviewed. Next, the domestic and foreign literature was reviewed for the contents of health information frequently encountered by late school-aged children. For this purpose, local health education textbooks for fifth and sixth graders in seven areas were analyzed; pamphlets related to child diseases and various health commons provided in elementary schools were also examined. As a result, the constituent factors in these documents were the ability to read and understand health-related documents [16] and to obtain health information [18], the comprehension of health-related communication, and the comprehension of health impact factors [12,14]. In this way, the factors of health literacy are presented in various ways. Recently, the concept of health literacy refers not only to the evaluation of the comprehension ability for health information, but also health-related communication. Therefore, it is necessary to develop a comprehensive tool for measuring health literacy, especially for children. However, considering the cognitive development of late school-aged children, the comprehensive health literacy component seems to be inappropriate for children. In addition, as suggested by a previous study [23], it was considered appropriate for children’s health literacy to focus on children’s formation of correct health habits and understanding of health information in daily life for a healthy life. Functional health literacy [24,25,26]—the most basic element of health literacy—is the ability to read and understand documents containing health information (reading comprehension) and numerical data (numeracy). Therefore, reading comprehension and numeracy were selected as constituent factors for developing a tool for measuring children’s health literacy ability.

#### 2.3.2. Development of a Preliminary Tool

The results of reviewing the preceding literature, to understand the content of health information for measuring children’s health literacy, are summarized as follows; general health [13,14]; physical activity, nutrition, diet, prevention of smoking [19]; and immunization and dental health [20]. Health information and terms included in the contents of the health curriculum were also examined. In addition, although the understanding of health information in the health system is the most basic constituent of health literacy measurement tools for adults, most of the children in the health care system are accompanied by their caregivers. Therefore, health information and terms contained in the content of the health curriculum pertaining to their daily lives were examined. Based on the results, four areas were selected: daily life and health, disease prevention and management, drug abuse, smoking and alcohol prevention, and accident prevention. Next, the research conducted in-depth interviews with four elementary school nurses and two elementary school teachers who are in charge of the daily life and health education of fifth and sixth graders. In this way, the most essential areas for the fifth and sixth graders in elementary school (i.e., “daily life and health”, “disease prevention and management”, and “drug abuse”) were selected. Based on the health information-related contents and terms included in the health education textbook for these 12 subdomains, 29 items were developed (Table 1). The examples and illustrations in the questionnaires used those in the textbooks that were examined. To prevent fixed responses from occurring, the questionnaires were prepared by distributing the items that measure the same concept. The preliminary tool consists of 29 questions in multiple-choice format with one correct answer and three incorrect answers.

#### 2.3.3. Expert Validity Verification

As the first step to verify the validity of the content of the preliminary tool, four professors in a nursing college examined the validity of the items, the necessity of additional items, the similarity between items, the arrangement of the linguistic expressions and the items, and the appropriateness of the items, and then revised them accordingly.

For the second step, 10 expert groups were formed; each group comprised four school nurses, two elementary school teachers, two professors of child nursing, and two professors of nursing, who had developed adult literacy measurement tools before, based on the number of experts for the validity of the content presented by Lynn [27]. After explaining the purpose of the study and the concept of children’s health literacy to the selected expert groups, the researcher distributed the prepared questionnaire for the preliminary tool, via e-mail, for content validation. According to Lynn’s criteria [27], the content of the question was rated as 4 points for “very valid”, 3 for “valid”, 2 for “not valid”, and 1 for “not at all valid”. The expert groups were also asked to provide opinions on questions that were ambiguous or unclear in meaning. The items with a Item-level Content Validity Index (I-CVI) value higher than 0.80 were selected. Among the 29 items, 5 items aimed at measuring health knowledge were deleted and reconstructed. Furthermore, a second content validity test was performed on the five newly developed items, and those with an I-CVI of 0.80 or higher were selected. The average of scale content validity index (S-CVI/Ave) was 0.89 (Table 1).

### 2.4. Ethical Consideration

This study was conducted after the institutional review board (IRB) had approved the study’s purpose, method, subject rights, and questionnaire (IRB No: 2014-02-010-001). A researcher visited each school, explained the purpose of the study and delivered the study guide, informed consent forms and questionnaires to the principal, homeroom teacher, and school nurse. The study guide describes the purpose of the study, the confidentiality of the response data and content, the ethical use of the study results, and that the subjects may opt not to participate in the study. The researcher and the homeroom teacher explained the study to the students and received the latter’s informed consent. Considering that the students are still underage, the researchers also distributed a notice to the students who agreed to participate to obtain their parent’s consent. The students who participated in the survey were provided with a gift in return.

### 2.5. Data Collection

The data collection period was from 11 May to 20 May 2014. The researchers visited each school. Because the the questionnaire contained correct and incorrect answers, the researchers asked the homeroom teacher and students to take precautions, such as not to discuss the question with each other at the time of taking questionnaire. The time required to complete the questionnaire ranged from 15 to 30 min, with an average of 24.7 min. The number of distributed questionnaires was 570; 559 were collected, with a return rate of 98.1%.

### 2.6. Data Analysis

Of the 559 returned questionnaires, 552 were used for final analysis, as seven were incomplete. All statistical analyses were performed using the IBM SPSS Statistics for Windows, Version 19.0 (IBM, co., Armonk, NY, USA), the BILOG-MG 3.0 (Scientific Software International, Inc., Skokie, IL, USA), and M-plus Base Program version 4.2 (MPLUS, Inc., Suwon, Korea). The concrete method of analysis is as follows: first, the content validity of the tool was verified by the I-CVI of the expert groups. Second, the internal consistency reliability of the tool was calculated as the KR-20 coefficient. Third, item analysis, construct validity, and criterion-related validity were assessed for construct validation of the instrument.

For the analysis of the items, the items were evaluated according to the classical test and item response theories. The exploratory factor analysis (EFA) was used for construct validity. For the estimation method, the maximum likelihood estimation and varimax rotation were used. In addition, we performed confirmatory factor analysis to assess the appropriateness of the classification of items measuring each variable using Mplus and to evaluate the suitability of the measurement model. To evaluate the criterion validity of the instrument, the health knowledge measurement tool, which was evaluated as a similar concept to health literacy, was used with the approval of the original developer. This was because the measurement tool for late school-aged students was not developed. The criterion-related validity was verified via the relationship with criterion tool. Fourth, Pearson’s correlation coefficient was calculated for validity of the criterion.

## 3. Results

### 3.1. Participants’ Characteristics

The subjects who participated in the evaluation of the Korean health literacy tool for late school-aged children included 552 fifth and sixth graders. With respect to their demographic characteristics, 53.2% of the participants were male and 46.7% were female. Overall, 52.1% were fifth graders and 47.8% were sixth graders. Regarding their place of residence, 44.9% subjects lived in a small city; 34.9% in a small town; and 20.1% lived in a big city. The most common type of family was nuclear, at 81.1%. The most common age of the father (77.1%) and the mother (67.5%) was 40 to 49. The next most common age of the mother was 30 to 39 (28.6%). The economic status of the children recognized by the children was “middle” (65.2%), followed by “good” (31.1%).

Among the health-related characteristics, 71.7% answered that they were healthy; 25.5% stated they were a little sick; and 2.7% were visiting the hospital regularly. In terms of their interest in health, 54.1% stated that their interest was “normal”, followed by 36.9% respobnding “a lot” and 8.8% stating “no interest”. In contrast, 67.2% of subjects stated that they thought their parents have “much interest” in their children’s health. When asked about the health information search path of the children, 69.7% said that they searched the Internet most, followed by 54.8% who asked “people around”, 38.7% who obtained information from “experts”, 33.7% who watched “TV”, and 31.8% who read “books”.

### 3.2. Validity

In order to verify the validity, the construct validity was analyzed through item analysis and exploratory factor analysis, and the number of constituent factors was verified and the validity of the criterion was verified through correlation with the reference tool.

#### 3.2.1. Item Analysis

The items were evaluated according to classical test and item response theories. First, the correlation between the total items and each item was analyzed. The correlation coefficient ranged from 0.14 to 0.57. Only items with a correlation coefficient between 0.30 and 0.80 were selected. No question yielded a coefficient value of 0.80 or more, whereas four items had a correlation coefficient of 0.30 or less. Therefore, the appropriate item number was 25 items. Second, the difficulty and discrimination of the items were calculated. The range of difficulty by item ranged from 0.55 to 0.76; the average difficulty of all items was 0.64. As the item difficulty was appropriate, no items were removed. In addition, the range of item discrimination ranged from 0.21 to 0.64, and the average discrimination of all items was 0.46. Third, the two-parameter logistic model (2PLM) was used to determine item difficulty (b) and item discrimination (a). According to this model, the total item difficulty (b) of the developed tool ranged from −1.88 to −0.34, indicating that most of the questions were easy to answer, whereas three questions were of medium difficulty. Item discrimination (a) ranged from 0.08 to 0.99. No item was thus deleted; no item had 0 or negative discrimination value (Table 2).

**Figure 1:**
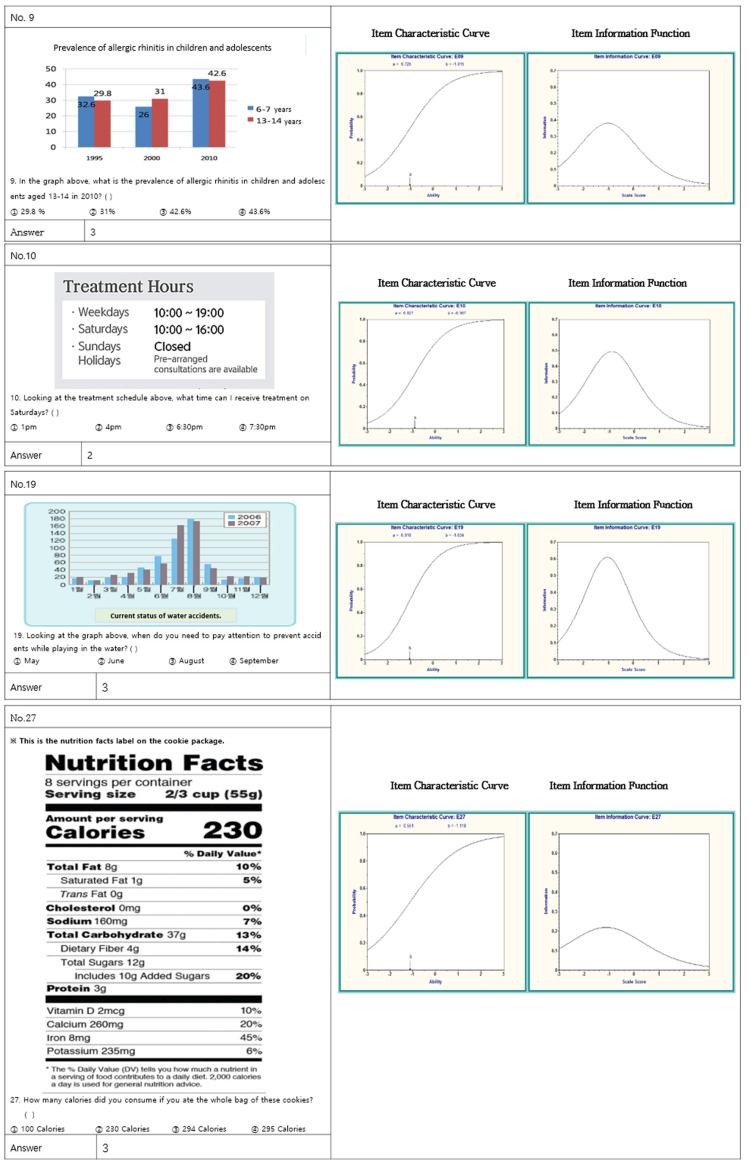
Examples of item characteristic curves (ICC) and item information functions (IIF).

**Figure 2:**
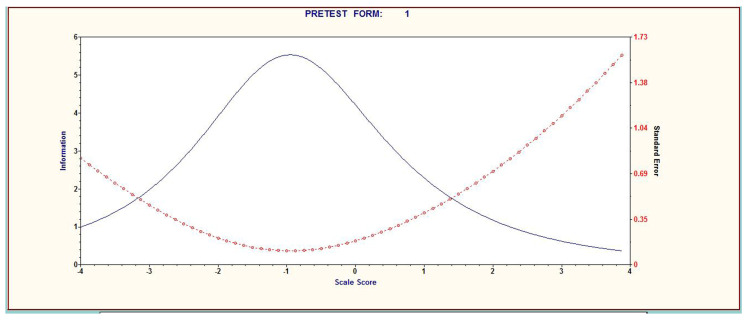
Graph of the item information function (IIF).

Finally, item analysis was carried out using the item characteristic curve (ICC), the item information function (IIF), and the test information function (TIF). ICC shows the potential of a subject to meet an answer for each item; IIF indicates the competent, appropriate persons to evaluate; and TIF is a set of ICC values of each item. TIF assumes 0 as the average level of ability. The developed tool was subsequently found to be a suitable tool for persons with ability of −1 (Figure 1 and Figure 2).

#### 3.2.2. Construct Validity

EFA was conducted to confirm the construct validity of the developed tool. Although there is no absolute criterion for factor loadings that indicate correlation with each item and factors, factors with a factor load of 0.40 or higher were extracted based on the assumption that a value of 0.30 or more is generally significant; conservative criteria use 0.40 or higher. In the first factor, the items with factor loadings of 0.40 or higher were items 9, 10, 14, 15, 17, 18, 21, 22, 23, and 28. Six items, namely items 5, 12, 16, 19, 26, and 27, were the second factor. The common attribute of the first factor was the ability to read and understand health related documents, which had an inherent value of 5.79. The common attribute of the second factor was the numerical ability required to understand health information, which had an inherent value of 1.99. Accordingly, the first factor was called “Comprehension of Documents”, or the ability to read and understand health related documents, whereas the second factor was the “Numeracy” required to grasp health-related information (Table 3).

In this study, the root mean square error of approximation (RMSEA), Comparative Fit Index (CFI), and Tucker–Lewis Index (TLI) values were computed to evaluate the model fit for the number of factors. An analysis of the components of the developed health literacy tool revealed that one to three factors can be predicted; thus, the number of factors was specified and analyzed. However, since χ^2^ values are sensitive to the sample size, we verified the model fit using RMSEA, CFI, and TLI values. Therefore, it can be seen that the model fit is most appropriate, when there are two factors (RMSEA = 0.06, CFI = 0.96, TLI = 0.95; Table 4).

#### 3.2.3. Criterion-Related Validity

To evaluate the criterion-related validity of the tool developed in this study, the health knowledge measurement tool, which is evaluated as a concept similar to the health literacy tool, was used. The primary reason for this use was the absence of a measurement tool for late school-aged students. The correlation between the developed tool and the health knowledge score analyzed was r = 0.62 (*p* < 0.001).

### 3.3. Reliability

The item-total score correlation (ITC) and internal consistency KR-20 reliability coefficient were evaluated to verify the reliability test of the final 16 items. ITC values ranged from 0.31 to 0.69, which was higher than |0.30| and satisfied the criteria [28], and all items reported a positive correlation. The internal consistency of the KR-20 reliability coefficient was 0.85, 0.88, and 0.82 for the overall tool, factor 1, and factor 2, respectively, which was above the reference value of 0.70 [29] (Table 3).

### 3.4. Final Item Selection

The final tool selected through the process of verifying the reliability and validity was composed of 16 items consisting of two components. This tool measures the health literacy of late school-aged children in a self-report format. It consists of four-choice items with the correct answer. The score range is 0–16; the higher the score, the higher the level of health literacy. The subjects in this study (i.e., 552 fifth and sixth graders) had a health literacy mean score of 13.16 (±2.24). Moreover, the correct response rate was 82.3%. Based on the evidence that health literacy depends on socioeconomic status [23], we confirmed the differences in health literacy scores according to general characteristics. There were no signficant differences according to the demographic characteristics of gender, grade, family type, father’s age, mother’s age, and household economic level. However, for residential areas, the total scores of students living in large cities was significantly higher (F = 27.9, *p* = 0.03). Similarly, there was no significant differences among the health-related characteristics of subjective health status perceived by children and the degree of interest in children’s health. However, significant differences were reported between regular hospital visits (t = 5.24, *p* < 0.001) and when parents were very interested in children’s health (t = 4.95, *p* < 0.001).

## 4. Discussion and Conclusions

The health literacy of children is important not only in terms of promoting their health in daily life but also in helping them become healthy adults with effective health promotion activities. However, research on the development of tools to measure children’s health literacy has not progressed in Korea and abroad. This study was thus attempted to develop a tool to measure the health literacy of late school-aged children.

As validation of the developed instruments, the study conducted item analysis, and evaluated both construct validity and criterion-related validity. First, four items with a correlation coefficient of less than 0.3, with respect to the total items, were found through item analysis; these items were subsequently omitted from the tool. These items were found to have low difficulty in item evaluation as well. Items that are too easy to evaluate are considered to have low contribution to and affect the stability of the tool; as such, care should be taken to maintain appropriate difficulty in tool development.

The item evaluation for difficulty and discrimination indicated that the difficulty of the final 16 questions according to the classical test theory was 0.66. The difficulty of the developed tool was evaluated as appropriate according to Seong’s evaluation criterion: the difficulty of a tool is appropriate when the difficulty is between 0.30 and 0.80 [28]. In addition, the item discrimination rate was 0.43; as an item is evaluated as a good item if the discrimination rate is 0.40 or higher, based on the classical test theory [28], the suitability of the developed tool was confirmed. However, the classical test theory evaluates an item using the total score of the item, leading to different results when used for different subjects and thus impeding generalizability [30]. Therefore, in this study, additional item evaluation based on item response theory was performed. An advantage of the item response theory is that the result of the item analysis does not change according to the target participant because the tool is analyzed by item, enabling estimation of the suitability of the ability item to the target person [28]. The difficulty (b) distribution of the developed tool ranged from −1.88 to −0.34, and according to the criteria presented by Seong [28], no item was extremely difficult (b > 2.0) or difficult (0.5 ≤ 0). Three items were evaluated as intermediate (−0.5 ≤ b < 0.5) in difficulty, and the remaining 21 items were easy (−0.5 ≤ b < −2.0). In addition, item discrimination (a) ranged from 0.08 to 0.99; four items had low discrimination (0 ≤ a < 0.34). The different results compared with the item evaluation according to the classical test theory were considered in light of the literature [31] indicating that the difficulty and discrimination of items based on classical test theory can be overestimated. Although the evaluation of difficulty and discrimination according to classical test theory is within the appropriate range; the difficulty level of each item includes items that are evaluated as easy. Additionally, differences in the basis of evaluation come into play: the questionnaire evaluation of classical test theory evaluates appropriateness based on the average difficulty or discrimination of the total items, whereas item evaluation according to item response theory evaluates the difficulty and discrimination scores for each item.

Apart from difficulty and discrimination, item response theory also analyzes the goodness of fit of items based on ICC, IIF, and TIF. TIF, which is a curve made by combining the IIF and ICC, was comprehensively analyzed to estimate the participants’ ability to be evaluated by the tool. The developed tool showed the best ability to identify persons with a performance level of −1 to 0. In other words, the tool was evaluated to be more suitable for children whose level of health literacy was lower than average, thereby matching the main purpose of the health literacy measurement tool: to select participants with low health literacy. Consequently, it can be regarded as a suitable tool for measuring the level of health literacy of children from low-income and multicultural families who are expected to have a low level of health literacy. In the future, the validity of the tool may be improved by examining the health literacy of children with various characteristics using this tool, and then comparing and analyzing the results of the item evaluation.

Factor analysis was then performed to confirm the construct validity. The two factors of “Document comprehension” and “Numeracy” were extracted through factor analysis. This finding is consistent with the two components derived from the literature review during the development of the tool, indicating that the theoretical composition of the tool is reasonable. Various scholars have suggested that “reading comprehension” and “numeracy” are basic attributes of functional health literacy skills [24,25,26] and basic components of health literacy measurement tools for children. Moreover, a study on Finnish students reported math and mother tongue grades to be important influencing factors [23], which is believed to support the current study findings. Thus, with the emerging need for “critical health literacy” to comprehend health information and choose better health habits, we propose the development of a “critical health literacy” instrument that accounts for the cognitive development of school age.

Next, the criterion-related validity was confirmed. The health knowledge tool, which was judged to be a similar concept, was used, given the lack of a developed tool for health literacy among late school-aged students. The r value of 0.62 was slightly higher than that (0.55) in An [6] for married migrant women. The criterion validity is reasonable, because the standard for reasonable criterion validity is between 0.40 and 0.60 [28]. The present results confirm those in a previous study [32], which defined health literacy as a comprehensive concept involving health knowledge. However, given the limitation of using health knowledge as a reference tool, the correlation validity between the health literacy scores measured by these tools and the children’s health literacy test tools needs to be verified.

Lastly, the internal consistency reliability of the tool was evaluated using KR-20 coefficients. The KR-20 coefficient of the final 16 items was 0.85, which is relatively high. Compared with developed tools for adults (considering the absence of tools for children), the internal consistency reliability coefficients of the tools developed in this study are reasonable. The KR-20 coefficients of developed instruments for adults are 0.98 for TOFHLA [33] and 0.76 for NVS [7]; for Korean version tools, KHLS [11], KHLI [7], and An [8] reported 0.89, 0.82, and 0.77, respectively. According to the criteria proposed by Nunnally [34], for a new tool, the internal consistency reliability is reasonable when it is 0.70 or more, and that of a mature tool is reasonable when it is 0.80 or more; the tool developed in this study thus has reasonable reliability.

Conclusively, 16 items were developed through validation and reliability tests. The developed tool was confirmed with reliability and validity, and then evaluated to be useful for late school-aged children whose level of health literacy was lower than average. The present study established validity through the evaluation of each item according to item response theory, which is known to generalize the characteristics of items and the ability of participants in a stable manner. In addition, Paakkari et al. [23] reported that the family’s economic status and gender were significant influencing factors. Moreover, this study reported significant differences according to the area of residence, regular hospital visits, and parents’ interest in children’s health. Therefore, we recommend future studies to identify factors affecting children’s health literacy, including health-related and various demographic and sociological characteristics, such as school grades and family’s economic status, which are considered to be influencing factors for children’s health literacy.

The results of this study suggest that the developed tool for health literacy measurement of late school-aged children will be useful, both in research and practice, to improve with proven validity and reliability the health literacy level of children. The data provided here are expected to contribute to a consensus on the conceptual definition of child health literacy. This study is limited due to the old data used in this research. However, health literacy has been reported to be a strong predictor for health promotion behavior. It enables accurate interpretation of health information and performance of appropriate health behaviors. Under these circumstances, this study demonstrates strength by developing a health literacy measurement tool for children. Moreover, although the data for the development of this tool are old, the tool was developed using the item response theory. Therefore, we have identified the unique properties of each item and have the capability to estimate the target’s ability to implement the tool.

## Figures and Tables

**Table 1:** List of KHLI-C from literature review and interview with school nurse and teacher.

Domain	Sub Domain	Item	Constituent Factor		I-CVI ^¶^	
			Document ^§^	Numbers ^‡^	1st	2nd
HDL *	(1) Health terminology (2) Physical activity	Health terminology	√		1.0	1.0
		Health terminology	√		0.7	0.8
		Health terminology	√		0.7	0.8
		Ambulatory treatment	√		0.8	0.8
		Clinic time table	√		0.6	0.9
		Medical department	√		1.0	1.0
		Safety accident graph		√	0.9	0.9
		Safety accident graph		√	0.7	0.8
		Physical activity	√		1.0	1.0
		Obesity prevention	√		1.0	1.0
	(3) Healthy food (4) Food sanitation (5) Food allergy (6) Healthy eating habits	Food Additives	√		0.8	0.8
		Food allergy	√		0.9	0.9
		Snack documentation	√		0.9	0.9
		Snack documentation	√		0.6	0.9
		Food composition table		√	0.8	0.8
		Food composition table		√	0.8	0.8
PM **	(1) Immunology and vaccination (2) Infectious disease (3) Scoliosis (4) Tooth decay prevention and management	Vaccination Precautions	√		0.8	0.8
		Cough manners		√	0.9	0.9
		IDP ****	√		1.0	1.0
		Scoliosis prevention	√		0.8	0.8
		Disease graph		√	1.0	1.0
		Tooth decay graph		√	1.0	1.0
		Dental caries description	√		0.8	0.8
DA ***	(1) Appropriate drug use (2) Smoking prevention	Dosage, usage instructions of drug	√		0.9	0.9
		Concept of drug abuse	√		0.9	0.9
		Concept of drug abuse	√		0.9	0.9
		Appropriate drug use	√		1.0	1.0
		Appropriate drug use	√		0.8	0.8
		Understanding non-smoking sign	√		1.0	1.0

*: Health and Daily Life, **: disease prevention and management, ***: drug abuse, ****: infectious disease prevention. ^§^: Health literacy expressed in text, ^‡^: health literacy expressed in numbers, ^¶^: Item-level Content Validity Index.

**Table 2:** Item analysis of instrument (*n* = 552).

Item Contents	Classical Item Response Theory (CTT)			Item Response Theory (IRT)		
	MFC *	α Value **	Difficulty	Dis ***	Difficulty	Dis ***
Health terminology	0.39	0.82	0.56	0.48	−0.49	0.70
Health terminology	0.39	0.82	0.55	0.56	−0.39	0.72
Health terminology	0.15 ^‡^	0.86				
What you need for hospital care	0.32	0.82	0.63	0.34	−0.89	0.40
Dosage, usage instructions of drug	0.57	0.82	0.61	0.49	−1.03	0.27
Reading the disease-specific graphs	0.49	0.81	0.66	0.35	−1.05	0.72
Concept of drug abuse	0.21 ^‡^	0.82	0.57	0.46		
Concept of drug abuse	0.22 ^‡^	0.86				
Understanding non-smoking sign	0.34	0.81	0.62	0.56	−1.02	0.73
Understanding Clinic timetable	0.38	0.81	0.58	0.33	−0.91	0.83
Understanding medical department	0.32	0.82	0.70	0.57	−0.76	0.67
Reading cavitation related graphs	0.32	0.82	0.61	0.55	−1.72	0.31
Understanding coughing manners	0.33	0.83	0.64	0.56	−0.57	0.99
Understanding food additives	0.33	0.81	0.76	0.21	−0.69	0.98
Scoliosis prevention	0.41	0.84	0.60	0.46	−1.02	0.90
Reading safety accident graph	0.40	0.81	0.75	0.26	−1.03	0.87
Vaccination Precautions	0.41	0.83	0.57	0.64	−1.36	0.08
Appropriate drug use instruction	0.49	0.81	0.76	0.47	−0.34	0.65
Reading safety accident graph	0.41	0.80	0.62	0.58	−1.03	0.92
Physical activity	0.36	0.82	0.64	0.49	−1.88	0.29
Obesity prevention	0.39	0.81	0.60	0.49	−0.73	0.52
Infectious diseases prevention	0.39	0.81	0.62	0.43	−0.55	0.51
School meals- food allergy	0.38	0.82	0.66	0.56	−0.60	0.54
Snack documentation-Expiration date	0.14 ^‡^	0.86				
Snack documentation-Induce allergies	0.33	0.82	0.67	0.44	−0.718	0.41
Food composition table	0.46	0.82	0.62	0.45	−1.561	0.21
Food composition table	0.34	0.81	0.70	0.47	−1.118	0.55
Understanding dental caries instruction	0.39	0.81	0.65	0.41	−0.954	0.74
Appropriate drug use instruction	0.39	0.81	0.64	0.46	−1.179	0.50
M ± SD			0.64	0.30		

^‡^ Question items with a correlation coefficient of less than 0.30. * Modified full correlation, ** α value if the item was removed, *** discrimination.

**Table 3:** Factor analysis of instrument (*n* = 552).

Item Contents	Factor Loading		KR-20
	Factor 1	Factor 2	Reliability Coefficient
Understanding Clinic timetable	0.71	0.38	
Scoliosis Prevention	0.68	0.41	
Understanding non-smoking sign	0.65	0.34	
Understanding dental caries instruction	0.56	0.39	
Understanding food additives	0.51	0.33	0.88
Appropriate drug use instruction	0.50	0.43	
Obesity prevention	0.48	0.39	
Vaccination Precautions	0.47	0.41	
Infectious diseases prevention	0.44	0.39	
School meals- food allergy	0.42	0.38	
Reading safety accident graph	0.41	0.72	
Safety accident graph	0.39	0.70	
Reading cavitation related graphs	0.41	0.48	0.82
Food composition table	0.41	0.46	
Food composition table	0.34	0.43	
Dosage, usage instructions of drug	0.38	0.40	
Eigen value	5.79	1.99	0.85

**Table 4:** Factor fit analysis (*n* = 552).

Factor	χ^2^ (df)	*p*	CFI	TLI	RMSEA
One Factor	142.02(51)	0.032	0.85	0.87	0.08
Two Factor	228.75(63)	0.002	0.96	0.95	0.06
Three Factor	77.96(40)	0.184	0.73	0.78	0.07

## Data Availability

The data presented in this study are available on request from the corresponding author.
